# Assessing the real benefits of surgery for degenerative lumbar spinal stenosis without instability and spondylolisthesis: a single surgeon experience with a mean 8-year follow-up

**DOI:** 10.1186/s10195-018-0497-8

**Published:** 2018-07-27

**Authors:** Riccardo Caruso, Alessandro Pesce, Valentina Martines, Venceslao Wierzbicki, Emanuele Piccione, Sergio Paolini, Tiziana Lanciano

**Affiliations:** 1 Dipartimento di Neurologia e Psichiatria, Sapienza University – Rome, Viale dell’Università 30, 00185 Rome, Italy; 2 A.O. “Sant’Andrea”, Neurosurgery Division, NESMOS Department, Sapienza University – Rome, Via di Grottarossa, 1035-1039, 00189 Rome, Italy; 3 Rome Army Hospital “Celio”, Piazza Celimontana, 50, 00184 Rome, Italy; 4 IRCCS “Neuromed”, Neurosurgery Division, Sapienza University, Via Atinense, 18, 86077 Pozzilli, IS Italy; 5 Department of Medico-Surgical Sciences and Biotechnologies, University of Rome “Sapienza”, Rome, Italy

**Keywords:** Lumbar spine, Lumbar spinal stenosis, Laminectomy, Iatrogenic instability, Lumbar spinal fusion, MRI

## Abstract

**Background:**

The degenerative lumbar spinal stenosis is one of the most commonly treated spinal disorders in older adults; despite its increasing frequency, it is not yet clear what the most effective therapy might be. The aim of this study is to investigate the very long term results of a homogenized cohort of patients suffering from lumbar spinal stenosis: the first subset of patients operated on with laminectomy and the second subset of patients was also advised to undergo laminectomy but never operated on.

**Methods:**

Patients from both subgroups were advised to undergo surgery, according to the same criteria, in the period between 2000 and 2010 and were re-evaluated in the period between January and December 2016.

**Results:**

Comparing the two subsets of patients, both suffering from clinically relevant LSS, the first subset returns a statistically significant clinical improvement at follow-up. The rate of excellent results decreases over years. Iatrogenic spinal instability incidence was found to be 3.8% in the present cohort.

**Conclusions:**

Although the improvement of the first postoperative years decreases over time and despite the lack of general consensus, the lack of established shared guidelines and the limitations of this research, the results support the utilisation of surgery for the management of this condition.

**Level of Evidence:**

3.

## Introduction

### Background and rationale

The degenerative lumbar spinal stenosis (LSS) is one of the most commonly treated spinal disorders in older adults [1–3] and, due to the aging of population, has become a widespread reason to undergo lumbar spinal surgery [1, 4]. It causes claudication, back and leg pain and disability [1–4]. Despite its increasing frequency, it is not yet clear what the most effective therapy might be [4].

Although high-impact trials [4] and prospective reports [5–9] in the relevant Literature suggest that surgery is the best choice over non-surgical treatments, data about long term follow-up are widely missing [7–9] as well as a precise algorithm concerning the correct choice among the different surgical procedures advocated, over years, for the management of this condition.

For this reason, we have decided to retrospectively analyse the experience of the first author of this manuscript to contribute to the research on LSS.

It is important to mention, however, that results from trials reporting a clear benefit from conservative treatment [10–12] have been published thus leading to the reappraisal of the role of sole conservative strategies for the management of this common condition, with results that are as good as those offered by surgical treatment.

### Objectives

First of all, the patients examined for this paper, have been treated rigorously by the same surgeon, this feature minimizes the possible bias related to a “technique” variability among different physicians, as far as personal and professional skills are concerned, which for a long time has been known to be a major bias factor, capable of jeopardizing the comparability of results [4, 13].

The aim of this work is therefore to re-explore this exceedingly investigated disease, to re-examine and question what is currently accepted about LSS. We, therefore, aimed at analysing only the *very long*-*term results* (7–16 years) of patients operated on with the classic surgical treatment for degenerative LSS (a facet-sparing laminectomy) versus a perfectly “matched” and comparable cohort of patients whose treatment, for various reasons, had been completely conservative and were never operated on.

## Materials and methods

### Study design and setting

The final cohort is composed of 112 patient**s** suffering from degenerative lumbar spine stenosis (LSS), surgically treated or listed for surgical treatment between January 2000 and December 2010, at the Neurosurgical Divisions of the IRCCS Neuromed Molise and later at the Rome Army Hospital; both the surgical and non-surgical subgroups of patients were personally evaluated and treated by the first author (R.C.) of this manuscript in his personal outpatient service in the same period (2000–2010).

### Participants and eligibility

All the patients included in the final cohort were diagnosed with LSS through a 1.5 T-MRI scan with T1 weighted and T2 weighted axial and sagittal scans without gadolinium. A standard high resolution CT scan of the lumbar spine to evaluate the contribution of “bony” elements in determining the stenosis was judged to be useful in many cases.

Furthermore, all the patients underwent a standard and dynamic X-ray study of the lumbosacral spine to assess whether there was any instability and none of the patients included in the final cohort was found to suffer from lumbar spinal instability at the time of surgical indication. We added a preoperative whole spine plain film as a routine radiological examination before surgery and no patients suffering from a major degenerative thoraco-lumbar scoliosis were included in the cohort.

Because of clinical history and neurological findings, all the LSS in this study had been referred for surgical treatment. Both subgroups shared the same clinical and radiological features according to the criteria used at that time:

1. Severe sagittal narrowing (less than 12 mm) of the spinal canal, caused either by “bony” or “ligamentous” hypertrophy, and/or compressive effect on nerve roots. This is an “a posteriori” criteria since we retrospectively noticed that such a severe narrowing was present in all cases, both in the non-surgical and surgical subgroups and even in the excluded cases (a total of 170 patients were operated on).
2. *Walking disability* the patients were unable to reach beyond 300 m and presented neurogenic claudication.
3. Motor or sensory mono or poli-radiculopathies detected with physical examination or with the aid of neurophysiology.
4. Invalidating lower-back pain lasting for more than 12 weeks, which improved with anterior flexion of the trunk or stable forward trunk posture.
5. When motor or sensory deficits had been absent or negligible and the major complaint had been the walking limitation, the patients had undergone a first line conservative treatment. Failure of this strategy had lead to surgical indication.

Both subgroups shared the same exclusion criteria:

1. No spinal instability was present in X-ray dynamic plain films,
2. No patient presenting gross spondylolisthesis was included in the final cohort,
3. No patient presenting gross scoliosis was included,
4. Lumbar spine sagittal balance was evaluated in all patients. In the final cohort were included only patients with a physiological lordotic lumbar curve.
5. All the patients included in the final cohort suffered from a purely lumbar spinal stenosis L1–L5.
6. In order to minimize the influence of the individual differences in the lumbar FSU degeneration, according to a previously published score [14] we included only patients with intervertebral disc that scored at least 3 or 4 according to Pfirrmann scale.
7. Patients with a history of previous lumbar, thoracic or cervical spine surgery, as well as patients with history of spine fractures, dislocations or spine inflammatory diseases were excluded because of the possibility that a previous spine surgery or condition could jeopardize the entire spine biomechanics and thus confound results.
8. Unavailable or incomplete surgical or clinical records,
9. Patients operated on (in surgical subgroup) presenting surgical complication such as epidural hematoma, surgical infections and incidental durotomy.
10. Patients who did not accept re-evaluation, dead, too sick or not attending the follow-up. When writing the present paper, we noticed that the exclusion of patients who did not accept revaluation could include patients who had an extremely good outcome with conservative management and did not feel a re-evaluation would be necessary and patients who experienced an extremely unsatisfying outcome or an unsatisfying medical relationship with the senior author (R.C.) in regards to the treatments proposed or actually performed. In our opinion, the possibility of re-evaluation refusals based on these very different circumstances create a form of bias, which can be controlled only with a study deriving from prospective recruitment of patients. It could be subsequently argued that our study design has investigated the “median”, and most represented, part of a ‘spectrum’ ranging from extremely unsatisfactory results to extremely satisfactory results in LSS management.

The selection of these particular exclusion criteria affected the number of patients eligible to be included in this study. Rather than include a larger sample with incomplete or partial data-collections and findings, it was felt that the comparability of the patients based on the clinical records was paramount and this was achieved by collecting the widest and most complete range of clinical data possible; rigid inclusion and exclusion criteria were meant to provide a clinically and anatomically homogeneous cohort of patients in regard to their specific lumbar spine condition, unconnectedly from the extra-spinal factors.

Psychosocial factors along with purely emotional factors could not be fully accessed and quantified and were therefore excluded.

The final cohort is composed of two subgroups.

The first subgroup of patients underwent simple laminectomy without fusion. To avoid nomenclature ambiguity, we term “laminectomy” a surgical procedure of spinosectomy, flavectomy and interspinosectomy with a bilateral complete sparing of the articular process. Discectomy and arthrectomy, as well as surgical fusion *were never performed* on the patients included in the study. Operative microscope was *routinely employed*. In case of foraminal stenosis, a foraminotomy was added to perform a nerve root decompression and appropriate adhesiolysis.

**Fig. 1:**
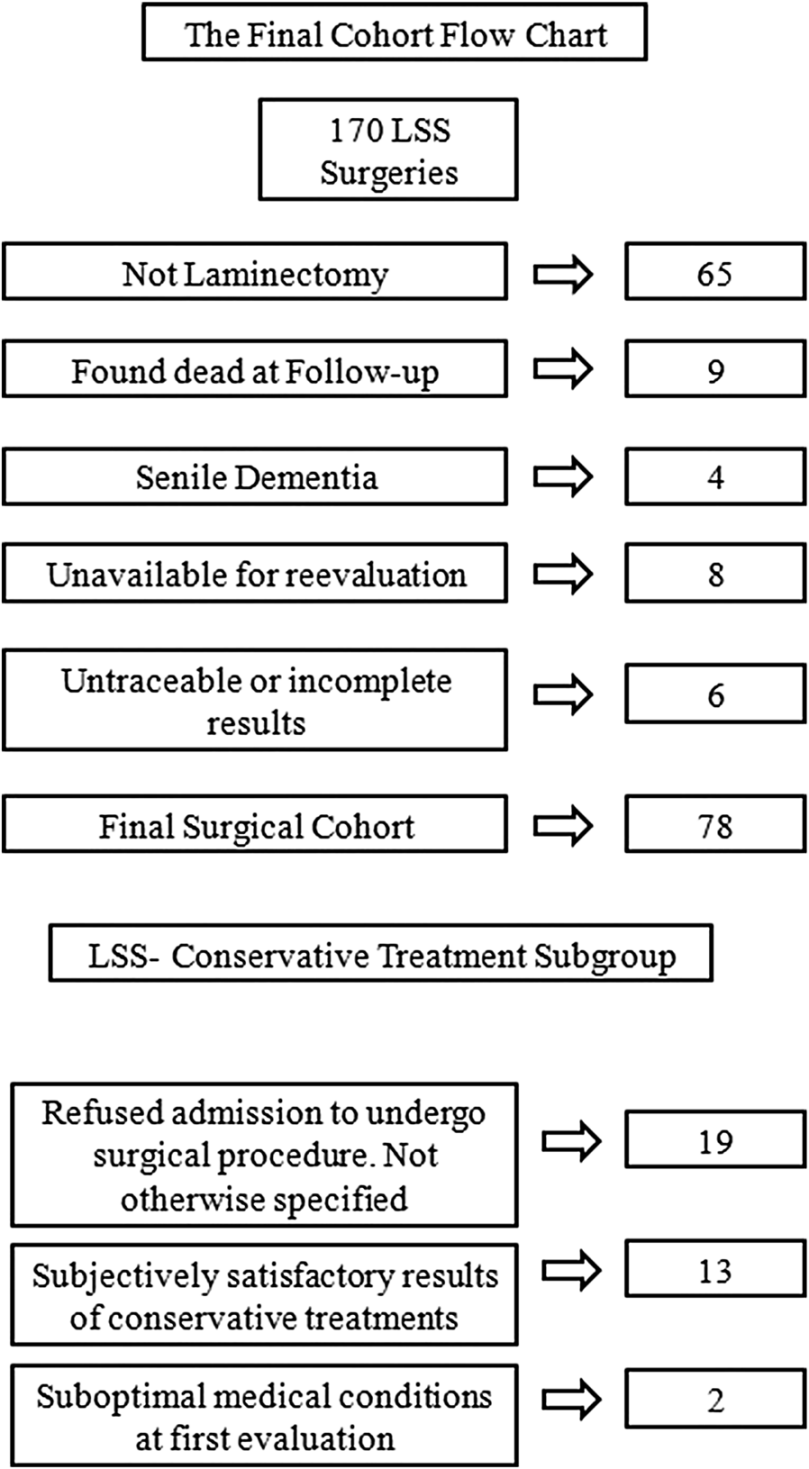
**a** Details of the surgical cohort. **b** Details of the non-surgical cohort

The second subgroup was composed of patients suffering from LSS who had been referred for a decompressive laminectomy according to the same aforementioned criteria. These patients, for various reasons (specified in Fig. 1) never underwent surgery and underwent instead conservative treatments composed of periodical physical therapy according to the Mézières technique.

The two subgroups of patients appear to be highly comparable according to the inclusion and exclusion criteria and on the ground of clinical course of LSS as a degenerative “clinical” condition, however it was not possible to access psychosocial and other lifestyle variables; thus comparability based on such data could not be assessed. The “anatomical” pattern cannot be regarded as comparable at follow-up because of the morphological differences induced by surgery among the different subgroups. Nevertheless, the aim of the study is to investigate the real impact of surgery in the management of the *clinical* impairment generated by this condition rather than to analyse the simple anatomical or surgical long-term results.

### Variables and data sources

Patients from both subgroups were contacted by phone, in the period between January and December 2016 to undergo the aforementioned follow-up lumbar spine imaging. When a patient, informed of the study design and purpose, accepted to undergo a radiological reevaluation, he/she underwent a clinical reevaluation with the aid of Swiss Spinal Stenosis Questionnaire (SSSQ). All the patients included in the study had been referred for surgery and/or underwent surgery in the period between 2000 and 2010 and were reevaluated between January and December 2016.

The SSSQ, is a self-report outcome tool, recommended by the North American Spine Society as the gold standard for the evaluation of the results after LSS surgery [15].

The Neuroimaging examination was performed by a team composed by a neuroradiologist and a neurosurgeon, who were kept completely in the dark as to the objectives of the study. This team was asked to judge if the craniocaudal and lateral extent of the laminectomy was enough, whether there had been a recurrence of LSS in the previously operated levels or LSS development at the higher or lower level; possible delayed iatrogenic vertebral instability was investigated as well.

The neurosurgeon was also asked to give out the SSSQ and to perform a complete neurological re-evaluation with a careful medical post-surgical history re-examination with attention not only to the data related to the stenotic disease that would affect the autonomy of the march and/or postural attitude, but also to the deterioration of other pre-existing conditions, such as osteoarthritis of the hip and knee, which can also contribute to the reduction of a patient’s walking ability.

Non-surgical subgroup completed a modified SSSQ: the last six questions referred to the outcomes of the physical treatment, rather than surgery.

After this evaluation, the team, supplemented by another neurosurgeon and a physiotherapist, then examined the SSSQs and according to the score, patients were divided into three categories: good (Score 18–38), moderate (Score 39–59) and poor (Score 60–79) outcome.

To avoid biases in the clinical re-evaluation process, the neurosurgeons who performed the clinical re-evaluation had not participated to any of the surgical procedures and never consulted the clinical records of the hospitalization before performing the neurological and general examination.

When the team completed both the radiological and clinical re-evaluation, clinical records from the first visit at the outpatient service (2000–2010) for both the surgical and non-surgical subgroups were compared with those gathered during the 2016 re-evaluation and then the clinical condition of each patients was ranked according to an ordinal three step variable as *improved, unchanged or worsened* compared to the preoperative state. In the last step, patients were asked to judge their condition in respect to the preoperative period or the outpatient clinical evaluation in which they received indication to undergo a surgical procedure.

### Missing data and potential sources of bias

The study cohort size has been dictated by the selection of the inclusion criteria. As previously stated there is no missing data since incomplete records was an exclusion criteria. On the post hoc estimated power tests, the size of the sample was found to be excellent (1 − *β*) = 0.986 (for *α* 0.05, effect size 0.8).

### Statistical methods

The entire cohort was analysed with SPSS v.18. ANOVA analysis was used to compare means of the scores between the subgroups. The variance of Age and Number of level treated, fully continuous and ordinal variables, have been split for statistical purposes and analysed as dichotomous variables. It is important to note that functional status and SSSQ outcomes, coded as previously described, could be considered as fully ordinal variables, provided with intermediate values. Bivariate correlation according to Pearson and Spearman were used for continuous and ordinal variables, Oneway ANOVA and Multivariate ANOVA were used to compare means between the two subgroups and between first evaluation and follow-up.

### Ethical and legal issues

All the patients of the surgical subgroup expressed consent to the surgical procedure after appropriate information was given. All the patients gave informed explicit consent to undergo the follow-up neuroimaging and clinical reevaluation, before performing the reevaluation they were elucidated about the purpose of the study. The local ethic committees of our Institutions had a favourable pronunciation about the ethical organization of the study because of its retrospective.

nature, because no treatment-randomization was performed and because MRI and plain X ray dynamic study imply minimal or no harm towards the individuals included in the final cohort and are included among the radiological worldwide gold standards for the follow up of LSS cases. Moreover, data reported have been completely anonymized.

This study is perfectly consistent, in any of its aspect, with WMA Helsinki declaration of human rights.

The study protocol approval relied on the following considerations:

1. MRI scans and X ray dynamic studies of LS do not deviate from the current unanimously accepted clinical practices concerning the radiological investigation of LSS;
2. LS-MRI scans were performed after written explicit informed consent of all the patients included in the final cohort.
3. LS-MRI scans were performed for free, patients did not undergo any additional fee and they were thoroughly informed about their radiological and neurological conditions after any follow-up MRI scan and they were not exposed to any kind of biological or psychological harm.
4. All the patients received a clear benefit from a free consultation by experienced spine surgeons and neuroradiologists after the MRI scan.
5. The benefits for society and for future patients are at the root of this study, which is aimed at gaining important conclusions about the effectiveness of decompressive surgery in treating LSS.

## Results

### Participants

Of a total of 170 patients operated on for lumbar stenosis and matching the aforementioned inclusion criteria, those who underwent simple laminectomy were 105, but we managed to complete the follow-up only to 78 of them; in fact, nine patients were found dead, other four were suffering from senile dementia, eight declared themselves unavailable to make a trip to undergo check-ups and all the remaining for various reasons have untraceable results.

The age of the 78 patients included in the surgical subgroup taken at the time of surgery ranged from 54 to 77 years, with an average of 66.6 years. There were 35 men and 43 women.

We managed to get in touch with 34 patients who were referred for decompressive laminectomy, but for various reasons were never operated on (Fig. 1). Among the 34 patients who matched the aforementioned inclusion criteria: 18 were males and 16 females; the age of the 34 patients tested at the time of first medical evaluation ranged from 52 to 78 years, with an average of 67.3 years.

The average follow-up was 8.2 years for both subgroups; the first surgical subgroup had an average follow-up of 7.9 ± 4.6 years and the nonsurgical subgroups of 8.2 ± 5.1 years with no statistically significant difference among the two subgroups. Thus, the final cohort including both subgroups amounted to 112 patients.

### Descriptive data

Table 1 summarizes symptoms and shows the levels of laminectomy. The average length of follow-up was 8.2 years with no statistically significant difference between the two subgroups. All the patients (both subgroups: the one that underwent surgery and that which didn’t) included in the final cohort presented an antero-posterior diameter of the spinal canal occluded to under 12 mm.

**Table 1:** Clinical disorders and presenting symptoms of the final cohort, operated levels of the surgical cohort, Swiss Spinal Stenosis Questionnaire for the final cohort and results of the team rating of the final cohort

Clinical disorders (%)	
Low back pain	88.8
Neurogenic claudication	74
Posture trunk forward	55
Lower limbs weakness	39
Monoradicular syndrome	26
Deficits of motion	22.2
Pluriradicular syndrome	16.6
Sensory disturbances	16.6

	Patients
Laminectomy level	
L1–L2	2
L2–L3	3
L3–L4	9
L4–L5	16
L3–L4/L4–L5	28
L2–L3/L3–L4/L4–L5	12
L3–L4/L4–L5/L5–S1	8
SSSQ results	
Outcome	
Surgical cohort	
Good	48 (61.5%)
Moderate	13 (16.7%)
Poor	17 (21.8%)
Non-surgical cohort	
Good	7 (20.6%)
Moderate	10 (29.4%)
Poor	17 (50.0%)
Team rating	
Results	
Surgical cohort	
Improved	54 (69.2%)
Unchanged	9 (11.5%)
Worsened	15 (19.3%)
Non-surgical cohort	
Improved	8 (23.5%)
Unchanged	12 (35.2%)
Worsened	15 (44.1%)

The neuroradiologist and neurosurgeon team examined the neuroimaging scans performed at the time of the first evaluation and the scans of the follow-up to see if there had been a latero-lateral or cranio-caudal progression of LSS or the presence of new iatrogenic vertebral instability.

Table 1 summarizes also the results of the SSSQ and the judgment of the team of authors who performed the re-evaluation concerning both the surgical and non-surgical cohort. Table 2 summarize the comorbidities capable of negatively affecting the walking ability found at follow-up.

**Table 2:** Comorbidities of the final cohort, both surgical and non-surgical cohorts

Disease	Surgical cohort	Non-surgical cohort
Comorbidities affecting the quality of the march		
Coxofemoral arthritis	3	1
Knee arthritis	2	1
Coxofemoral arthritis + knee arthritis	2	
Cervical spondylotic myelopathy	4	1
Chronic heart failure	1	3
Varicose veins—lower limbs	5	2
Arterial occlusive disease—lower limbs		1

In the subgroup who underwent surgery, in specific regards to patients affected by comorbidities (Table 2):

- 8 patients were classified as “worsened” and “poor outcome”,
- 2 patients were classified as “unchanged” and “poor outcome”,
- 4 patients were “unchanged” and “moderate outcome”,
- 3 patients were “improved” and “moderate outcome”.

Among the non-surgical subgroup all those affected by comorbidities (as shown in Table 2) were judged as “worsened” and “poor outcome”. Table 3 summarizes the outcome of the neuroradiological imaging findings. Among the patients who had been operated on, 11 presented the following situation at follow-up:

**Table 3:** Relevant neuroimaging findings of the final cohort

Neuroimaging findings	
Surgical cohort	
Recurrence of the stenosis in the intervention area	2
Recurrence of stenosis in a higher area than the intervention	1
Recurrence of stenosis in a lower area than the intervention	1
Insufficient laminectomy in craniocaudal direction	4
Vertebral instability	3
Non-surgical cohort	
Worsening stenosis	3
Vertebral instability	1

- 8 showed an unsatisfactory outcome of the laminectomy, including vertebral iatrogenic instability and recurrent LSS; they were classified as “worsened” and “poor outcome”,
- 1 patient presented an insufficient craniocaudal extension of the laminectomy and was classified as “unchanged” and “moderate outcome”,
- 1 patient presented modest vertebral instability and was classified as “improved” with “good outcome”,
- 1 patient presented a recurrent LSS at the level under the previously operated one and was classified as “improved” with “good outcome”.

We then submitted all data obtained from the study to a statistical enquiry with SPSS v. 18 software.

### Statistical inference: outcome data and main results

There was no statistically significant difference in the age and length of follow-up between the two subgroups (*p* .470 and *p* .516, respectively). Age, both considered as a continuous and considered as a dichotomic variable (> 60 or < 60 years old) was not related for both subgroups to the number of levels affected or treated (respectively, *p* .304 and *p* .748).

**Fig. 2:**
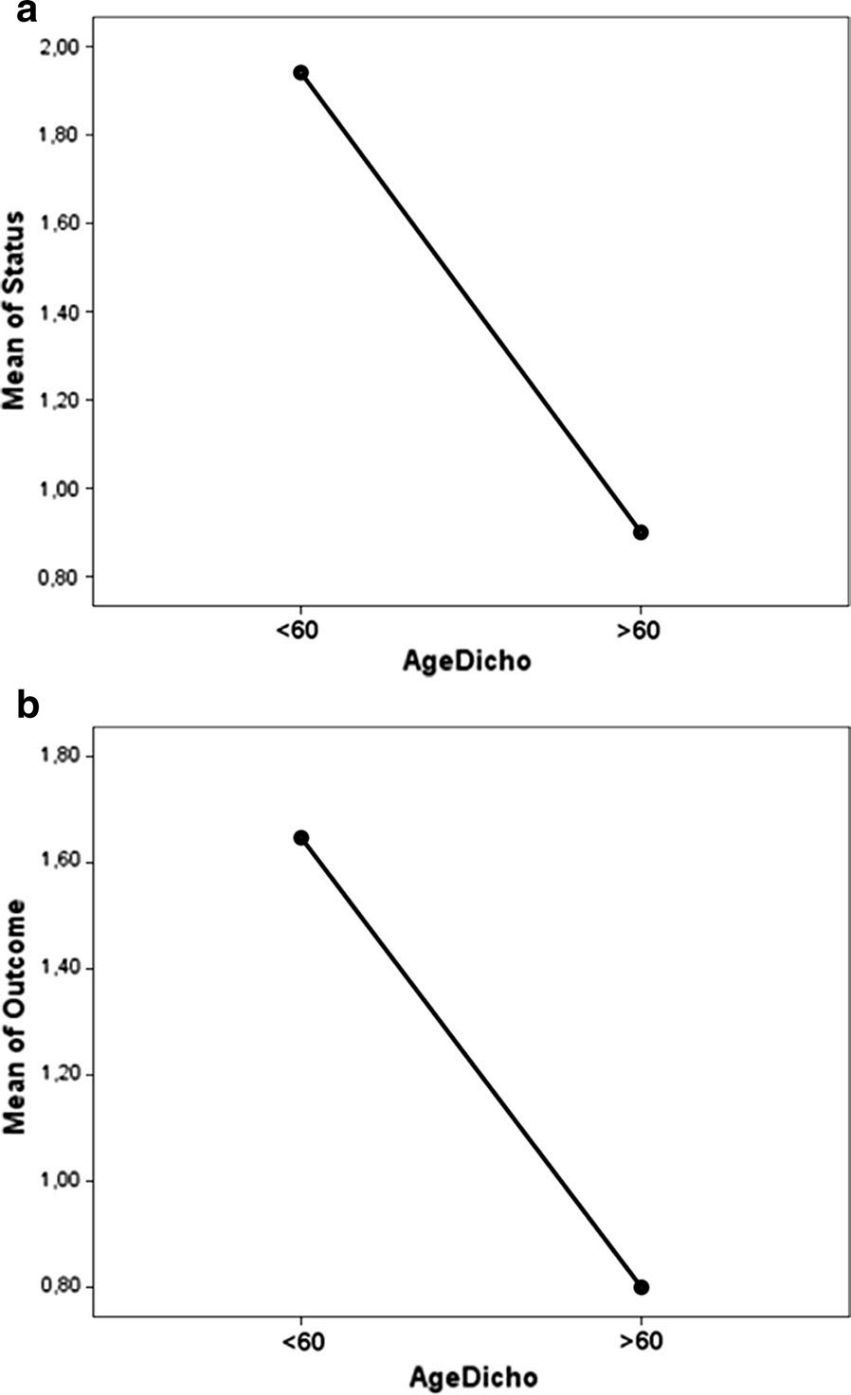
One-Way ANOVA analysis showing the statistically significant difference between patients older and younger than 60 years old in regards to **a** functional status and **b** SSSQ outcomes

Conversely, average age of the *surgical subgroup* showed a strong negative statistical relation both with functional status and with SSSQ outcome (respectively, *r* = − .559 with *p* .001 and *r* = − .420 with *p* .019). These results were absolutely confirmed both for SSSQ outcome and functional status when considering age as a dichotomic variable (respectively *p* .001 and *p*. 008, Fig. 2a, b).

Functional status at follow up presented a high relation with SSSQ outcome as evaluated by team rating at follow-up (*r* = .821 with *p*. > 0001). Functional status and SSSQ outcomes were not related with the number of levels treated in the surgical cohort (respectively, *r* = − .118 with *p* .279 and *r* = − .210 with *p* .146). Conversely a trend toward statistical significance was found for the same parameters in the non-surgical subgroups (respectively *r* = − .418 with *p* .088 and *r* = − .210 with *p*. 106), demonstrating a lesser efficiency of the conservative treatment for the multilevel LSS.

### Other analyses

**Fig. 3:**
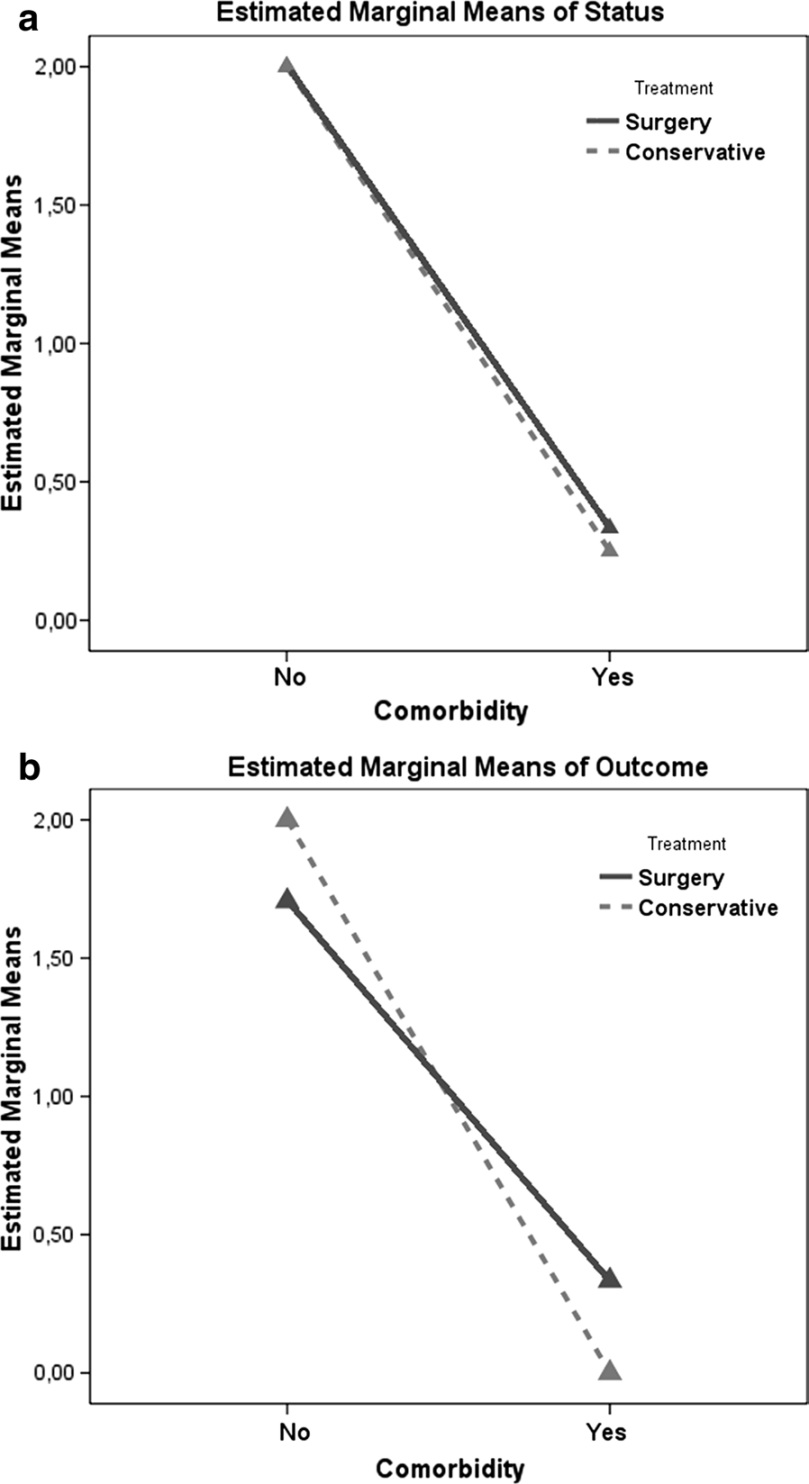
Multivariate ANOVA analysis showing the statistically significant worse. **a** Functional status and **b** SSSQ outcomes for patients presenting comorbidities mentioned in Table 1, regardless of the treatment performed

We found a strong statistical impact of the presence/absence of comorbidities affecting the quality of the march both on functional status and on outcome as defined by the SSSQ (both *p* > .0001) irrespective of whether conservative or surgical treatment had been carried out (*p* .151 and *p* .737 respectively; Fig. 3a, b), demonstrating the great clinical impact of these conditions on long-term results. Among the comorbidities, coxofemoral osteoarthritis showed the strongest statistical association with a “poor outcome” (*p* > .0001). The two different subgroups presented no statistically significant difference in regards to the incidence of comorbidities (*p* .378).

## Discussion

The aim of this study was to retrospectively analyse the very long term outcomes of an *ultraselected, bias*-*free* cohort of patients operated on by one surgeon, for a simple lumbar central canal stenosis, and to compare these results to those of a cohort of patients who were found to suffer from a perfectly superimposable condition at first outpatient evaluation and who, according to the same surgical indication criteria, were referred for surgical treatment but, due to various reasons, were never operated on.

In all the patients included in the final cohort, no spinal instability, no gross sagittal imbalance or degenerative spondylolisthesis were present: in fact all the patients included suffered from LSS with an antero-posterior narrowing of the spinal canal to under 12 mm, which is currently recognized as a “threshold” in the recent relevant Literature [16, 17].

This feature was shared both by the surgical and non-surgical subset of patients.

We pragmatically accepted this as a post hoc radiological parameter among many others reported in the Literature as it featured in all the cases [16]; we used this parameter to judge if the extension of the decompression after the procedure was satisfactory or to detect LSS relapses. The radiological features for the evaluation of LSS widely lack standardization and consensus [16].

Obstacles to this work lie in the multifactorial aetiology of LSS [17–19]: if we consider only the anatomical conditions, it is possible to notice that single factors like lumbar spondylarthrosis, lumbar spine instability, facet malposition and hypertrophy, the different degrees of intervertebral discs degeneration, hypertrophy or calcification of the posterior ligamentous complex (PLC) and anatomical variations of the spinal canal diameter may play roles of a dramatically different weight in different patients affected by a *similar* form of LSS.

### Key results and Interpretation

As previously reported [20, 21], the rate of excellent outcomes for surgical treatment over long periods (many years), decreases by about 70%, and this data appears to be confirmed in the present series.

However a detailed and critical review of our data mitigates the disappointment that might initially rise from the long-term results of surgery in the management of this condition.

There was a statistically significant difference between the “self report” outcomes (SSSQ) and the clinical and radiological outcomes as seen from the physician point of view: patients were definitely older when reevaluated at follow up and many of them suffered from other degenerative conditions affecting the *quality* of their march. Patients did not appear to be completely aware of how much of their reduced physical efficiency could exclusively be attributed to their LS conditions.

In four patients (about 5% of the surgical subgroup), the cranio-caudal extension of the laminectomy had been insufficient, thus results were unsatisfactory; this common surgical complication significantly contributes to the failure of the procedure; at present, with the constantly improving quality of intraoperative imaging, such “failures” can be simply avoided.

In three patients the laminectomy caused delayed lumbar spine instability, significantly contributing to a bad outcome. Two of these patients were later operated on for the newly arisen instability and LS fusion was performed, the remaining patient was deemed unsuitable for surgery and thus referred for physical treatment. Therefore, in the present series, the incidence of iatrogenic spinal instability over a span of 12–17 years was 3.8%, which is not negligible, though not unacceptable.

In the present series, patients underwent a standard laminectomy (“partial laminectomy” with sparing of the medial facet of the articular process). We strongly prefer to spare articular processes because of the evidence-based risk of iatrogenic postoperative instability. In the thoracolumbar spine, instability usually appears in 25% of patients receiving more than 2 level laminectomies if the articular processes are involved in the osteotomies [21]. Postoperative deformity is reported in 9.4% of patients receiving complete laminectomies compared to 3% in patients whose articular processes are preserved [22]. When the preoperative imaging rules out the presence of a gross preoperative lumbar spine instability, a laminectomy without fusion can be safely performed, with minor risk of jeopardizing spinal stability [23].

Avoiding a useless spinal fusion leads to a reduction in surgical and anesthesiological times, intraoperative bleeding, spinal stiffness, iatrogenic neurological and spinal adverse events related to an unnecessary procedure and furthermore, in countries like Italy, in which the National Health System provides full coverage for healthcare expenses, leads to a dramatical case related cost reduction for patients operated on for LSS [23].

A judicious follow-up, properly hastened in case of painful symptomatology and/or neurological variations, brings to a fast and effective detection of instability; in such cases, when the dural sac and neuroforamina have been previously decompressed, a minimally invasive lumbar arthrodesis can definitely resolve the problem.

If we compare the surgical and the non-surgical subsets of patients, it appears that the management of LSS through surgery, despite several limitations, is the most effective treatment for this condition. Despite some evidence that in the long-term surgery presents the same outcomes of the conservative strategy [7, 24], our results confirm the majority of previous reports [4–9]: in the long run, patients who undergo surgery preserve better neurological and functional status in respect to non-operated patients.

As previously stated, we investigated the very long term results of a classic surgical technique for the management of LSS: the decompressive laminectomy, but, at present, there are other minimally invasive techniques that are increasingly being used for LSS surgery. Obviously, every procedure that minimizes handling and damage of the tissues, reduces bleeding and reduces the length of surgeries is welcome, but currently we lack strong evidence, large and multicentric prospective trials with very long term follow-up to accurately estimate the effectiveness of the new procedures.

### Limitations and generalisability

The main limitations of this study lie in the exiguity of the sample and in its retrospective nature.

Bias are expected from the long range of follow-up, the lack of access to complete psychosocial and other lifestyle factors and paradoxically even from the inclusion and exclusion criteria: the selection of the patients enrols a limited number of highly comparable patients on one side, but on the other may exclude potentially relevant observations since, as reported in the Literature, psychosocial and emotional factors do influence the expression of impairment caused by degenerative lumbar spine conditions in the quality of life [25–28].

Furthermore, while a single surgeon’s experience study depicts what is the real story of a single operator in the management of LSS, and importantly eliminates technique-related bias that may develop among different operators, it may on the other hand exclude relevant observations about the general history of this disease, as well as psychosocial variables that may have affected the operator himself. Though a great effort has been made towards a rigorous methodology in patient’s eligibility, conclusions may suffer from under-representation bias, and prospective randomized large cohorts of patients, treated by a select number of surgeons, are required to provide conclusive findings in regard to the management of this condition. It would be particularly interesting to run at least two parallel, comparative studies on the prospective sample: one that, like ours, takes only take into consideration the anatomical and clinical factors, the other that includes the psychosocial and emotional variables.

What makes this study relevant is that the impact of many types of common bias has been completely removed. Moreover, one the greatest bias, the technique related difference among different physicians has been excluded because all the patients have been operated on by the same surgeon.

The increasing incidence of LSS, its impact on quality of life and on treatment related costs for National Health Systems, compels researchers to urgently propose evidence-based guidelines for the management of this condition. The road to reach this target is not easy because of the large number of confounding factors that must be taken into account.

Surgery appears to be the most effective treatment and the current gold standard for the management of LSS.

## Abbreviations

LSS: lumbar spinal stenosis
LS: lumbar spine
CT: computed tomography
MRI: magnetic resonance imaging
SSSQ: Swiss Spinal Stenosis Questionnaire
